# Effects of Kinesio tape on individuals with carpal tunnel syndrome: a randomized controlled study

**DOI:** 10.3389/fresc.2024.1494707

**Published:** 2024-11-08

**Authors:** Wei-Han Chen, Willy Chou, Min Hsu, Yu-Lin You, Yu-Lin Wang, Yuan-Yang Cheng, I-Ting Lui, Chuan-Ching Liu, Lan-Yuen Guo

**Affiliations:** ^1^ Department of Sports Medicine, College of Medicine, Kaohsiung Medical University, Kaohsiung, Taiwan; ^2^ Department of Physical Medicine and Rehabilitation, Taichung Veterans General Hospital, Taichung, Taiwan; ^3^ Physical Medicine and Rehabilitation, Chi Mei Medical Center, Tainan, Taiwan; ^4^ Rehabilitation Medicine, Chi Mei Medical Center, Tainan, Taiwan; ^5^ Department of Sports Medicine, China Medical University, Taichung, Taiwan; ^6^ Ph. D. Program in Biomedical Engineering, College of Medicine, Kaohsiung Medical University, Kaohsiung, Taiwan; ^7^ Department of Medical Research, Kaohsiung Medical University Hospital, Kaohsiung, Taiwan; ^8^ Center for Long-Term Care Research, Kaohsiung Medical University, Kaohsiung, Taiwan; ^9^ College of Health Sciences, Kaohsiung Medical University, Kaohsiung, Taiwan; ^10^ Research Center for Precision Environmental Medicine, Kaohsiung Medical University, Kaohsiung, Taiwan; ^11^ College of Humanities & Social Sciences, National Pingtung University of Science and Technology, Pingtung, Taiwan

**Keywords:** carpal tunnel syndrome, Kinesio tape, pain intensity, hand grip strength, electroneurography

## Abstract

**Objective:**

Carpal tunnel syndrome (CTS) is a common neuromuscular disorder with an incidence rate of 4.9%. Research on the impact of Kinesio taping (KT) on electroneurography in individuals with CTS is limited, highlighting a significant gap in the literature. This study aimed to evaluate the effects of KT on palm numbness, pain intensity, hand grip strength, and median nerve electroneuromyography in individuals with mild to moderate CTS.

**Method:**

This was a randomized, controlled, parallel design study. Twenty-seven participants diagnosed with CTS through hospital outpatient clinics were randomly assigned to either the control or KT group. The outcome measurements included hand grip strength, the Boston Carpal Tunnel Questionnaire (BCTQ), and electroneurography, assessed at baseline and after 6 weeks of intervention. The statistical method used was non-parametric analysis, comparing differences with the median and interquartile range (IQR).

**Results:**

Both the KT and control groups significantly improved the numbness grades, hand grip strength, and the BCTQ. The kinesio-taping group demonstrated an improvement in grip strength with a median increase of 2.21 kg (IQR: 0.65–3.79 kg), compared to the control group, which showed a median increase of only 0.70 kg (IQR: 0.22–1.45 kg). Statistical analysis revealed a significant difference between the two groups (*p* = 0.039), with an effect size of *r* = 0.33.

**Conclusion:**

This study suggested that KT can be a supplementary treatment to relieve pain intensity, enhance hand grip strength, and improve sensory conduction velocity, motor latency, and motor amplitude.

**Clinical Trial Registration:**

https://doi.org/10.1186/ISRCTN82192319, ISRCTNregistry (No. ISRCTN82192319).

## 1 Introduction

The incidence of carpal tunnel syndrome (CTS) is 4.9%, with a higher incidence in women (1). Repetitive hand movements are a risk factor for CTS, with a 5.4% prevalence in those working over an hour daily, compared to 1.8% in those who do not (2). CTS symptoms include pain, numbness, dysesthesia, and, in severe cases, hand muscle atrophy (3). It is caused by median nerve compression and may lead to paresthesia, grip weakness, and thumb muscle atrophy, affecting daily hand function (4, 5). CTS can affect one or both hands, with studies showing that 60%–70% of cases are bilateral (6). A nerve conduction examination is used to diagnose numbness caused by cervical nerve compression from CTS. Mild to moderate CTS is diagnosed when distal sensory latency exceeds 3.5 ms (4).

Clinically, CTS can be treated with surgery or conservative methods, with conservative treatment typically recommended for mild to moderate cases (7). Common conservative treatments are the use of non-steroid anti-inflammatory drugs (NSAIDs) and physical therapy, such as ultrasound, wax therapy, laser, splint, and so on (7, 8). The goals of the conservative treatments were to relieve pain and functionality restoration, and these conservative treatments have been reported to have some positive effects on pain relief and numbness (9–13).

Kinesio tape is applied to the skin to facilitate muscle contraction, increase blood flow, and activate analgesia to relieve pain (14). Recently, Kinesio tape has been used for symptom relief in individuals with mild to moderate CTS (15–17). Previous studies indicated that Kinesio taping may be more effective in the long-term investigation of hand grip strength (16). Hence, Kinesio taping may be a supplementary treatment for individuals with CTS. However, these claims were based on theoretical assumptions and require further research to confirm their effectiveness. The efficacy of Kinesio tape in reducing pain, increasing strength, and providing sensory stimulation is inconsistent across studies (18–20). There are few studies using electroneurography to examine the effectiveness of KT on individuals with CTS.

Thus, this study aimed to investigate the effects of the Kinesio taping on pain intensity, disability levels, hand grip strength, and electroneurographic parameters in individuals with mild to moderate CTS. This study hypothesized that the pain intensity, hand grip strength, wrist function, nerve conduction velocity, and motor latency would be improved after Kinesio taping intervention; in addition, the extent of difference of pre- and post-measurements was greater after Kinesio taping intervention compared to the conservative therapy only.

## 2 Method

### 2.1 Experimental design

This randomized controlled design study evaluates participants diagnosed with CTS. Each affected hand was assessed as a separate unit. Participants were randomly assigned to either the Kinesio tape group or the control group using a lottery system. In cases where a patient had CTS in both hands and met the inclusion criteria, random allocation was carried out separately for each hand. The lots were drawn by an assistant who did not participate in the study. Outcome measurements were taken at baseline and after a 6-week intervention. A physician who did not participate in the intervention program handled the group assignment process, and a physical therapist carried out all taping interventions. The study was approved by the institutional review board of Taichung Veterans General Hospital (approval number CF20351A), and the trial has been registered on the ISRCTN registry (no. ISRCTN82192319).

### 2.2 Participants

**Figure 1:**
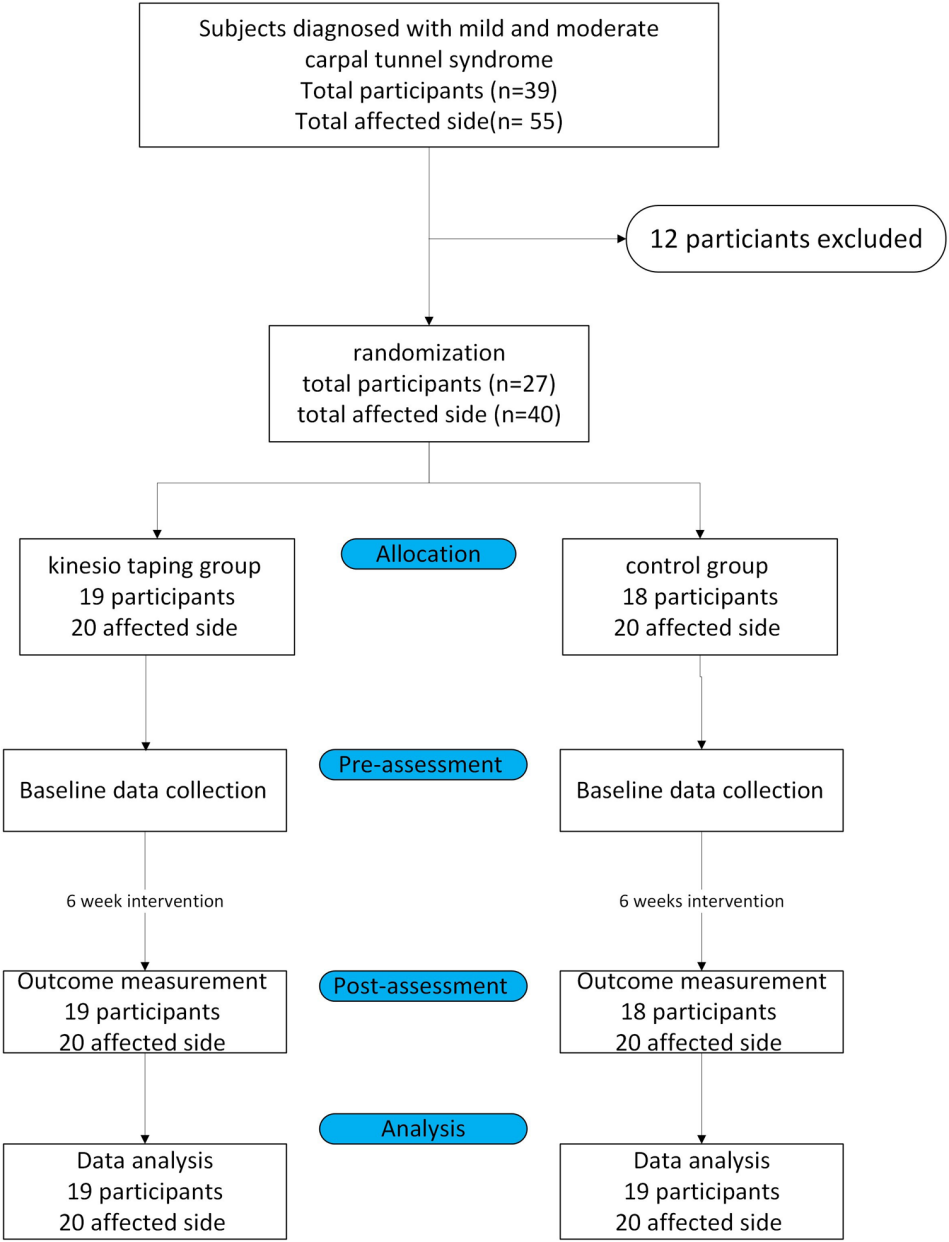
The study flowchart.

Thirty-nine individuals with CTS were evaluated for eligibility; two refused to participate, nine could not cooperate with the complete experiment, and one withdrew due to skin allergy discomfort. Hence, a total of 27 individuals with CTS participated in this study. Investigators explained the experimental procedure before data collection. The participants signed informed consent forms before participating in this study. The inclusion criteria of this study were: (1) participants aged 18–65 who were diagnosed with mild to moderate CTS by a physician, and (2) CTS symptoms persisted for at least 3 months. The exclusion criteria were: (1) severe CTS diagnosed by a physician and atrophy of the palm muscles, (2) steroids injection on the wrist in the past year, (3) skin status was not suitable for Kinesio tape, such as psoriasis was allergic, experienced inflammation or had open wounds, and (4) a history of surgery on the wrist. All participants only received the interventions at the research clinic and did not receive any other form of treatment. They were also instructed not to use the affected wrist to lift objects weighing more than one kilogram and to avoid repetitive wrist movements that could make symptoms worse. The study flowchart is shown in Figure 1.

### 2.3 Treatment sessions

#### 2.3.1 Kinesio-taping group

**Figure 2:**
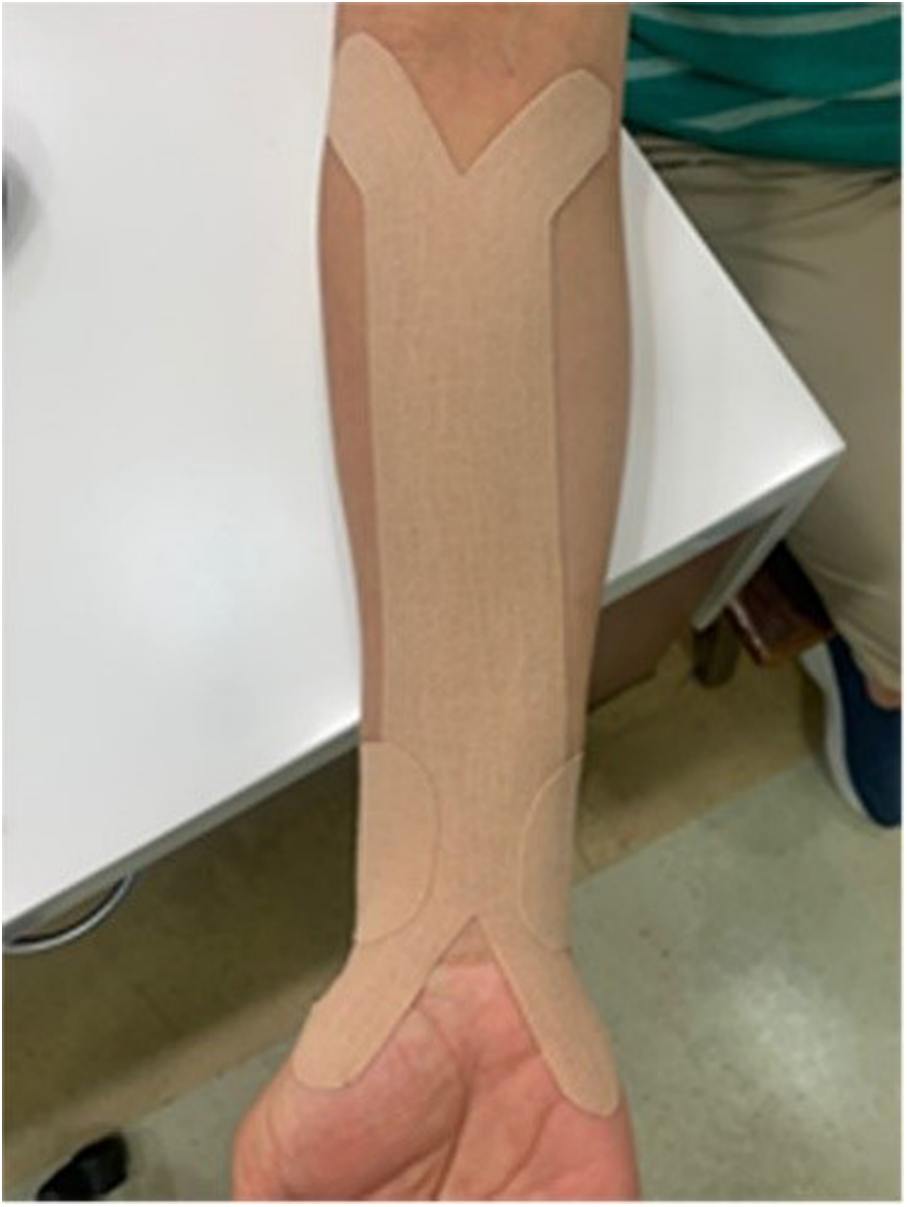
Kinesio taping application.

In the kinesio-taping group, in addition to conservative physical therapy, the Kinesio tape (SKT-X-050, JAPAN, 50 mm × 4.6 mm) was applied to the forearm (from the elbow joint to the wrist joint) twice a week for 6 weeks, and the tape was kept on the taping site for 2 days. In one intervention session, two Kinesio tapes were applied to a participant. Participants were asked to extend their wrists at 30 degrees with forearm supination. The x-shape Kinesio tape was first applied to the mediolateral epicondyles tension-free. Then, the Kinesio tape was attached through the forearm with a slight tension (15%–25%) to the first and fifth metacarpophalangeal joints without tension. The second Kinesio tape was an I-shape tape. After the center point of the sticker was attached to the dorsal side of the distal radioulnar joint, the two ends adhered to both sides of the distal radioulnar joint with a slight tension (15%–20%) (17) (Figure 2).

#### 2.3.2 Control group

Participants in the control group received conservative physical therapy, including heat therapy, transcutaneous electrical nerve stimulation, ultrasound, and laser treatment twice a week for 6 weeks.

### 2.4 Outcome measurements

The outcome measurements of this study include pain intensity, hand grip strength, nerve conduction velocity, and the severity and disability levels of the affected hand. The outcome measurements would be evaluated at the baseline and after intervention.

#### 2.4.1 Pain intensity

The visual analog scale is a highly reliable tool for evaluating subjective pain intensity (10, 16, 21). Participants were requested to rate their subjective pain perception from 0 to 10 on a 10-centimeter scale. Zero represents pain-free, while score 10 represents severe pain (22).

#### 2.4.2 Hand grip strength

The electronic hand grip strength dynamometer (TTM, YDD-110, JAPAN) was used to evaluate hand grip strength. Participants performed three grip strength tests with elbow full extension, with a 10-second interval between each test. The average of the three grip strength measurements was used to determine the parameter.

#### 2.4.3 Boston carpal tunnel questionnaire

The severity and the disability levels of the hand were evaluated by the Boston Carpal Tunnel Questionnaire (BCTQ), a high-reliability questionnaire to assess the severity of CTS symptoms and disability levels (23). The Boston Carpal Tunnel Syndrome Questionnaire consists of the Boston Functional Status Scale (BCTQ-Functions) and the Boston Symptom Severity Scale (BCTQ-Severity). The Functional Status Scale primarily measures the degree to which symptoms affect daily activities, while the Symptom Severity Scale assesses the severity of symptoms caused by the condition. The higher scores on this questionnaire represented more severe symptoms and more movement difficulty (23, 24). The previous studies found good face, content, and construct validity as well as test-retest reliability (*r* = 0.91) and internal consistency (0.80–0.90) on the BCTQ- Severity scale as well as high test-retest reliability (*r* = 0.93) and internal consistency (0.88–0.93) on the BCTU-Function scales (24, 25).

#### 2.4.4 Electroneuromyography

In this test, the nerve conduction velocity and latency were measured by a physician and evaluated by dual-channel nerve conduction electromyography (ENMG) (Nicolet VikingQuest-Natus, USA). The evaluation was conducted at room temperature between 24°C and 26°C and with a hand skin temperature higher than 30°C. The motor distal latency, motor amplitude sensory distal latency, median nerve sensory amplitude, and sensory nerve conduction velocity of the abductor pollicis muscle were recorded.

### 2.5 Statistical analysis

IBM SPSS 20.0 was used for statistical analysis. Baseline parameters of quantitative data were presented as arithmetic mean ± standard deviation (SD). The Shapiro-Wilk test was used to assess the normality of quantitative variables. The Wilcoxon Signed Rank Test was used to assess differences between pre-test and post-test measurements within both groups, with outcome measures presented as median and interquartile range (IQR). Additionally, the Mann-Whitney *U* test was used to compare differences between the pre-test and post-test groups. A significance level of 0.05 was used for all statistical tests. Effect sizes (*r*) were calculated to quantify the magnitude of the differences, with *r* values of 0.1 indicating a small effect, 0.3 a medium effect, and 0.5 a large effect. Effect sizes were calculated using the formulas provided by the previous research, which offers comprehensive tools for effect size computation and transformation (26).

## 3 Results

A total of 27 CTS participants (22 female, 5 male, 40 hands in total) aged 30–65 years were included in the study. There were 13 participants with a diagnosis of CTS in both hands, and 14 participants had CTS in one hand only. All participants were right-hand dominant. The mean age of the kinesio-taping group was 49.8 ± 8.47 years old, and the mean age of the control group was 47.2 ± 3.17 years old. The participants were all right-hand dominant. The outcome measurements at the baseline were not significantly different between groups (Table 1).

**Table 1:** The comparisons of baseline measurements between groups.

Parameters	Control group	Kinesio taping group	*p*-value	
	Mean (SD)	Mean (SD)	Mann-Whitney *U* test	Shapiro-Wilk test
Age (year)	47.2 (3.17)	49.8 (8.47)	0.695	0.232
Gender female, *n* (%)	15 (83)	17 (89)	0.782	0.713
Wrist affected side, *n* (%)				
Right	13 (65)	14 (70)	0.940	0.650
Left	7 (35)	6 (30)		
Pain intensity (score)	5.15 (1.73)	5.45 (2.24)	0.320	0.000*
Hand grip strength (kg)	19.27 (2.28)	22.55 (6.96)	0.270	0.000*
BCTQ- Severity (scores)	22.15 (5.46)	20.92 (6.99)	0.650	0.042*
BCTQ-functions (scores)	13.08 (4.99)	10.83 (4.15)	0.098	0.000*
Motor distal latency (ms)	4.97 (1.32)	4.70 (1.10)	0.936	0.134
Motor amplitude (mV)	7.42 (2.97)	7.29 (2.88)	0.538	0.328
Distal sensory latency (ms)	3.99 (1.23)	4.17 (1.75)	0.769	0.007*
Sensory amplitude (mV)	16.11 (8.74)	16.37 (8.42)	1.000	0.000*
Sensory conduction velocity (m/s)	34.92 (8.85)	33.33 (10.22)	0.728	0.135

BCTQ, Boston Carpal Tunnel Questionnaire. The Shapiro-Wilk test was used to assess the normality of quantitative variables.

* *p* < 0.05.

After 6 weeks, both the control and the kinesio-taping groups demonstrated significant improvements in pain intensity, hand grip strength, severity of symptoms, and function movement during daily activities (Table 2). In the nerve conduction velocity evaluation, both groups showed significant improvements in motor amplitude before and after the intervention (Table 3). The kinesio-taping group significantly improved distal sensory latency and sensory conduction velocity, particularly in the non-dominant hand. The control group exhibited statistically significant differences in sensory conduction velocity, motor distal latency, and motor amplitude with the dominant hand (Table 3).

**Table 2:** The comparisons of the outcome measurements between baseline and after a 6-week intervention within both groups.

Parameters	Control group		*p*	*r*	Kinesio taping group		*p*	*r*
	Baseline	Post			Baseline	Post		
	Median (IQR)	Median (IQR)			Median (IQR)	Median (IQR)		
Pain intensity (scores)								
Total	5.00 (4.00–6.00)	2.00 (1.00–3.00)	<0.001**	0.81	5.50 (3.50–7.00)	2 (0.25–3.00)	<0.001**	0.81
Right side affected	5.00 (5.00–7.00)	2.00 (1.00–4.00)	0.008**	0.81	6.00 (5.50–8.50)	2.00 (0.00–3.00)	0.007**	0.89
Left side affected	4.00 (3.50–5.50)	2.00 (1.00–3.00)	0.011*	0.84	5.00 (3.00–6.00)	2.00 (1.00–3.00)	0.011*	0.76
Hand grip strength (kg)								
Total	20.82 (17.95–22.38)	21.75 (19.15–24.77)	0.004*	0.64	18.89 (17.32–22.67)	21.87 (19.56–25.98)	<0.001**	0.80
Right side affected	20.03 (17.20–21.30)	20.70 (17.00–22.00)	0.026*	0.67	19.60 (17.32–24.65)	24.66 (20.43–33.35)	0.008**	0.87
Left side affected	22.56 (19.61–30.54)	23.10 (20.69–31.21)	0.066	0.61	18.87 (17.3–21.16)	21.03 (17.9–22.00)	0.021*	0.70
BCTQ- Severity (scores)	19.50 (16–25.5)	13.50 (12.25–16.75)	<0.001**	0.80	21.00 (15.25–29.5)	13.50 (11.25–15.75)	<0.001**	0.86
BCTQ-functions (scores)	9.00 (8.00–14.75)	8.50 (8.00–10.75)	0.002*	0.69	11.00 (8.00–15.00)	8.00 (8.00–9.00)	0.003**	0.66

The Wilcoxon signed-rank tests were used to test the differences between pre- and post-measurements within groups. *r*, effect size. IQR, interquartile range; BCTQ, Boston Carpal Tunnel Questionnaire.

* *p* < 0.05.

** *p* < 0.01.

**Table 3:** The comparisons of the electromyography parameters between baseline and after a 6-week intervention for both groups.

Parameters	Control group		*p*	*r*	Kinesio taping group		*p*	*r*
	Baseline	Post			Baseline	Post		
	Median (IQR)	Median (IQR)			Median (IQR)	Median (IQR)		
Motor distal latency (ms)								
Total	4.50 (4.48–9.13)	4.20 (3.17–5.19)	0.002*	0.68	4.43 (4.02–5.01)	4.32 (3.62–5.51)	0.695	0.09
Right side affected	5.00 (3.56–5.92)	4.74 (3.13–5.21)	0.008**	0.80	4.43 (3.96–4.90)	4.53 (3.86–5.34)	0.128	0.51
Left side affected	4.35 (3.53–5.24)	4.12 (3.19–5.05)	0.086	0.57	4.43 (4.01–6.04)	3.87 (3.52–5.73)	0.229	0.36
Motor amplitude (mV)								
Total	7.50 (4.48–9.13)	7.74 (6.72–10.85)	0.04*	0.47	6.96 (5.01–7.65)	9.01 (6.65–10.00)	0.003**	0.66
Right side affected	6.70 (4.00–9.20)	6.80 (4.20–10.10)	0.197	0.39	6.10 (4.95–9.95)	9.90 (6.05–10.60)	0.062	0.77
Left side affected	8.02 (4.99–10.40)	8.21 (7.30–11.30)	0.139	0.49	5.92 (4.91–7.50)	8.30 (6.60–9.70)	0.021*	0.56
Distal sensory latency (ms)								
Total	3.42 (2.65–4.62)	3.32 (2.6–4.17)	0.081	0.39	3.65 (3.10–4.61)	3.06 (2.05–4.11)	0.009**	0.58
Right side affected	3.28 (2.71–5.47)	3.19 (2.86–4.32)	0.445	0.23	3.54 (2.96–3.94)	2.55 (2.01–4.06)	0.263	0.37
Left side affected	3.56 (2.58–4.31)	3.44 (2.16–3.80)	0.069	0.61	3.72 (3.18–4.74)	3.13 (2.12–4.27)	0.010*	0.78
Sensory amplitude (mV)								
Total	13.29 (8.97–21.53)	14.18 (10.3–24.33)	0.247	0.26	16.62 (11.60–23.40)	16.56 (13.35–24.56)	0.108	0.34
Right side affected	11.00 (8.20–14.20)	12.10 (8.38–15.5)	0.534	0.20	15.16 (8.70–21.65)	15.10 (9.75–22.10)	0.173	0.44
Left side affected	15.20 (11.46–29.26)	23.50 (13.98–27.66)	0.374	0.30	19.15 (11.90–24.20)	17.00 (14.00–28.00)	0.374	0.27
Sensory conduction velocity (m/s)								
Total	35.50 (28.5–45.75)	35.50 (31–48.75)	0.020*	0.52	36.00 (28.25–40.50)	37.00 (30.50–49.50)	0.007**	0.61
Right side affected	35.00 (26.00–45.00)	35.00 (30.00–47.00)	0.05	0.59	35.00 (28.50–40.50)	35.00 (27.00–50.50)	0.326	0.32
Left side affected	42.00 (31.00–50.50)	38.00 (32.50–52.50)	0.158	0.47	36.00 (27.00–41.00)	39.00 (30.00–48.00)	0.006**	0.83

The Wilcoxon signed-rank tests were used to test the differences between pre- and post-measurements within groups. *r*, effect size; IQR, interquartile range.

* *p* < 0.05.

** *p* < 0.01.

**Figure 3:**
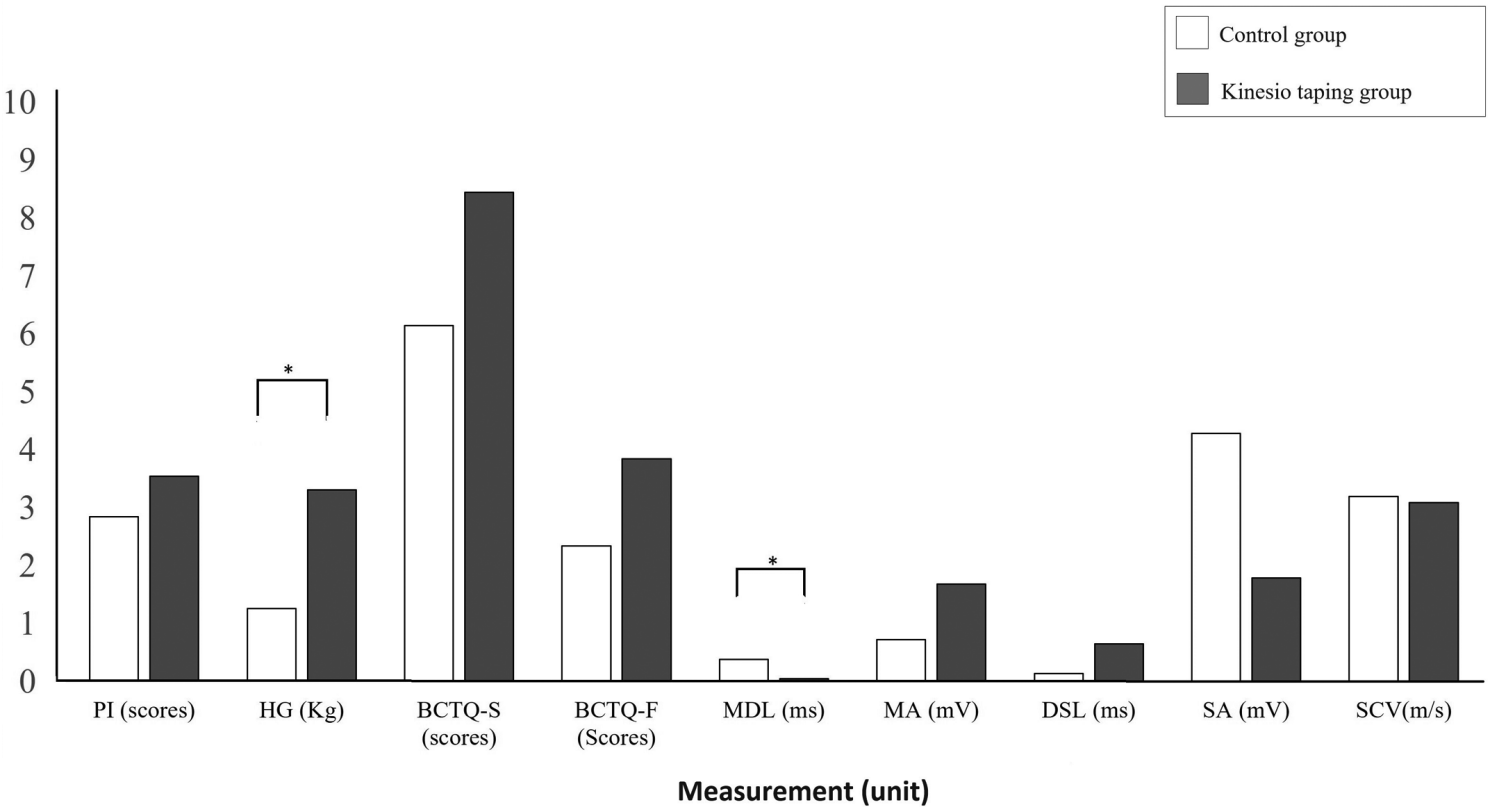
The comparisons of improvement extent between the control group and the kinesio taping group. PI, pain intensity; HG, hand grip strength; BCTQ-S, Boston Carpal Tunnel Questionnaire-Severity; BCTQ-F, Boston Carpal Tunnel Questionnaire-Function; MDL, motor distal latency; MA, motor amplitude; DSL, distal sensory latency; SA, sensory amplitude; SCV, sensory conductive velocity; *p < 0.05 with Mann-Whitney *U* test.

The improvement extent between the kinesio-taping group and the control group revealed that the kinesio-taping group demonstrated a significant improvement in hand grip strength difference relative to the control group (median in the kinesio-taping group: 2.21 kg with IQR: 0.65–3.79 kg; control group: 0.70 kg with IQR: 0.22–1.45 kg; *Z* = −1.012, *p* = 0.039, *r* = 0.33), while the control group demonstrated a significant improvement in the motor distal latency difference compared to the kinesio-taping group (median in the kinesio-taping group: 0.00 ms with IQR: −0.45 to 0.19 ms; control group: −0.47 ms with IQR: −0.65 to −0.07 ms; *Z* = −2.070, *p* = 0.038, *r* = 0.33) (Figure 3).

## 4 Discussion

The results of this study showed that both groups experienced significant improvements in hand grip strength and median nerve electroneuromyography after the intervention. However, the combination of conservative physical therapy and Kinesio tape led to greater improvements in hand grip strength compared to conservative physical therapy alone. On the other hand, conservative physical therapy was more effective in improving motor distal latency than Kinesio tape. Regardless of the intervention group, both approaches were effective in improving CTS-related pain, electrophysiological performance, and hand function.

Previous indicated that there was a significant association between pain intensity and disability level (27). Pain may cause fear and avoidance of performing movements and further result in disability. In the current study, both kinesio-taping and control groups decreased pain intensity, and the BCTQ significantly improved disability level and severity. The control and kinesio-taping groups received modalities such as ultrasound, laser, TENS, and heat therapy. A possible explanation for the decrease in pain intensity in both groups might be that ultrasound may reduce afferent pain input by increasing the stimulation threshold, possibly through hyperpolarization, which likely relieves pain by decreasing pain perception. Because of that, the Kinesio tape applied on the pain site reduced the chemical receptors induced stimulation, which may relieve the pain intensity and improve circulation (28). The Kinesio tape stimulated the mechanoreceptors to activate gate theory to reduce pain intensity (15).

In the current study, both groups showed significant improvements in hand grip strength compared to the baseline, with the kinesio-tape group achieving a significantly greater level of improvement than the control group. This result was consistent with our hypothesis that the hand grip strength improved after the Kinesio tape intervention. Kinesio tape was thought to facilitate tendon movement via neurological inhibition (29). The increased hand strength and pain relief observed following the intervention resulted in a reduction in the symptomatic expression of carpal tunnel syndrome and a corresponding improvement in the subject's functioning in daily life, as evidenced by the improved results on the BCBQ. Furthermore, the kinesio-tape group's significant improvement in hand grip strength was also mirrored in the ENMG results for motor amplitude, which reflected the nerve's ability to activate muscle fibers and the overall state of the nerve-muscle connection. Motor amplitude reflected the ability of the nerve to activate muscle fibers and the response of the muscle to the innervated nerve, indicating how many motor units were activated and representing the overall state of the nerve-muscle connection (30). Kinesio Tape, applied from the origin to the insertion of the muscle at a tension of 15%–25% of its original length, enhanced muscle contraction (31), thereby promoting an improvement in hand grip strength.

The electrophysiological criteria for the CTS patients included reduced nerve conduction velocity (<50 m/s) and/or prolonged motor latency (>4 ms). The electrophysiological diagnostic criteria for mild CTS included prolonged sensory or mixed distal latency, with or without amplitude reduction, while moderate CTS was characterized by these same features in addition to prolonged median motor distal latency (32). In the ENMG results of this study, the control group showed significant improvement in motor distal latency compared to the kinesio-tape group. Although the statistical comparison between the two groups did not reach a significant difference, the control group had more severe symptoms in both aspects of the BCTQ scale and motor distal latency compared to the kinesio-tape group. Motor distal latency assessed the transmission speed of nerve signals and the integrity of the nerve's myelin sheath. Delayed motor distal latency was often observed in cases of myelin damage or nerve compression and was one of the criteria for determining moderate CTS (32). The control group underwent conservative physiotherapy, including treatments such as heat, ultrasound, TENS, and low-level laser therapy, to directly improve circulation to the affected area and deliver nutrients necessary for nerve repair. These treatments helped reduce pain and symptoms or improve function in mild to moderate CTS in the short to midterm (15, 20). Although Kinesio tape was an effective treatment for improving function and reducing pain, the ENMG results suggested that its effectiveness in promoting neural repair, particularly in terms of motor distal latency, may have been lower than that of direct application of physical modalities to moderate CTS in short term intervention.

In the current study, the kinesio-taping group demonstrated significant improvements in the sensory conduction velocity compared to the baseline measurement. The increased amplitude of the neurons and the conduction velocity represented the increase in excitability (33). Sensory fibers are more susceptible to ischemic change because they contain a higher proportion of large myelinated fibers, which require more energy to function compared to motor nerve fibers. As a result, sensory fibers are more likely to be damaged from compression earlier than motor fibers (34). Besides, applying the Kinesio tape decreased the stress and pressure on soft tissues and nerves, which may further reduce tension and increase proprioception (28). On the other hand, the sensory changes occurred before the motor changes (35). This phenomenon likely accounts for why participants in the kinesio-taping group demonstrated more remarkable improvement in distal sensory latency after the intervention compared to the control group.

Specifically, an improvement in ENMG results for the left hand was observed in the kinesio-taping group, indicating an increased muscle response. This improvement was likely due to improved motor nerve input or, to a lesser extent, a reduced degradation resulting from repetitive strain. The non-dominant hand might be less affected by demyelination or axonal damage, which is common in overused limbs (36). These findings suggested that treatments to enhance nerve conduction benefit the non-dominant hand. The superior recovery observed in the non-dominant as evidenced by the ENMG results in the kinesio-taping group, might be attributed to a complex interplay of factors, including reduced initial damage, diminished continuous strain, and potentially enhanced responsiveness to interventions like Kinesio taping due to its lower baseline stress and strain levels (37). The non-dominant hand was used less in daily activities, especially for work or household tasks, where the dominant hand was primarily employed. Reducing the frequency of use may have been one of the factors that helped the nerves recover and reduce inflammation. In addition, the use of Kinesio Tape increased blood circulation, which accelerated nerve transmission and promoted recovery.

### 4.1 Study limitations

There were some limitations to this study. Firstly, there is a lack of follow-up evaluation after treatment is completed. A previous study reported that both the low-level laser intervention and the combination of the low-level laser and the Kinesio tape demonstrated significant improvement in pain intensity, disability level, and hand grip strength after a 3-week intervention; however, after completing the intervention, only the low-level laser and the kinesio-taping group demonstrated significant improvement on the hand grip strength in CTS individuals (16). Conservative physiotherapy combined with Kinesio tape or conservative physiotherapy alone both improved CTS symptoms in the short term, but this study only investigated a 6-week intervention. However, due to the lack of follow-up, it remained uncertain how long these improvements were maintained. Future research should include a follow-up period to assess the long-term effectiveness of short-term interventions and to better understand the time effects of Kinesio taping after the intervention. For instance, a crossover study design for patients with bilateral CTS could be implemented, incorporating an appropriate washout period and follow-up duration to record the sustained effects of the interventions.

Several potential biases and errors could have affected the validity and reliability of results when conducting a study in which both experimental and control groups were applied to the same participant. A major limitation was the lack of a placebo in the control group, which reduced the ability to minimize potential bias. To better isolate the effect of Kinesio tape, it would be methodologically stronger to use a placebo tape without therapeutic properties that mimic the Kinesio tape application. In addition, the crossover effect was another significant limitation. Treatments applied to one hand could have affected the condition of the other, making it difficult to separate the effects of each treatment, as psychological expectations played a role, although each hand was treated as an independent unit for evaluation. Participants might have had different expectations for each treatment applied to their hands, influencing their subjective symptom reports. This expectation bias could have biased the results to reflect the participants’ beliefs rather than the treatments’ effectiveness. Interdependence was another issue. Treating both hands of the same participant meant interventions worked within the same physiological system. Changes or improvements, on the one hand, could have had neurological or behavioral effects on the recovery, thus confounding results.

To mitigate these interdependence biases more effectively, the study design required stricter randomization, consideration of physiological and psychological crossover effects by applying a placebo, crossover trial design with a proper washout period, and larger sample sizes. In addition, it was essential to control the order of treatments and ensure that both raters and participants were as blinded as possible to the allocation and treatment process. These measures helped increase the reliability and validity of the study results.

## 5 Conclusion

This study demonstrated that both conservative physical therapy and Kinesio Taping were effective in improving pain intensity, hand grip strength, electrophysiological performance, and hand function in people with CTS after a short-term intervention. The combination of Kinesio tape and conservative physical therapy resulted in significantly greater improvements in hand grip strength and sensory conduction velocity compared to conservative therapy alone, while conservative therapy was more effective in improving motor distal latency. Both groups showed improvements in BCTQ scores, reflecting reductions in disability and symptom severity. These findings suggest that Kinesio Tape may be a complementary treatment for short-term improvement in pain, handgrip strength, and conduction velocity in CTS.

## Data Availability

The raw data supporting the conclusions of this article will be made available by the authors, without undue reservation.
